# Clinical verification of the relationship between serum lipid metabolism and immune activity in breast cancer patients treated with neoadjuvant chemotherapy

**DOI:** 10.1186/s40001-022-00964-w

**Published:** 2023-01-02

**Authors:** Wataru Goto, Shinichiro Kashiwagi, Koji Takada, Yuka Asano, Kana Ogisawa, Tamami Morisaki, Masatsune Shibutani, Hiroaki Tanaka, Kiyoshi Maeda

**Affiliations:** 1 Department of Breast Surgical Oncology, Osaka Metropolitan University Graduate School of Medicine, 1-4-3 Asahi-Machi, Abeno-Ku, Osaka, 545-8585 Japan; 2 Department of Gastroenterological Surgery, Osaka Metropolitan University Graduate School of Medicine, 1-4-3 Asahi-Machi, Abeno-Ku, Osaka, 545-8585 Japan

**Keywords:** Breast cancer, Tumor immune activity, Lipid metabolism, Neoadjuvant chemotherapy

## Abstract

**Background:**

Lipid metabolism has been recently reported to affect the prognosis and tumor immune activity in cancer patients. However, the effect of lipid metabolism on chemosensitivity in patients with breast cancer treated with neoadjuvant chemotherapy (NAC) remains unclear.

**Methods:**

We examined 327 patients with breast cancer who were treated with NAC followed by curative surgery. The correlations between the serum levels of total cholesterol (TC) and triglyceride (TG) and the clinicopathological features, including the efficacy of NAC, neutrophil-to-lymphocyte ratio (NLR), and absolute lymphocyte count (ALC), were evaluated retrospectively.

**Results:**

Serum TG levels were increased after NAC in all the subtypes, and the rate of change was the highest, especially in triple-negative breast cancer (TNBC) (21.0% → 48.1%). In addition, only TNBC patients with an objective response (OR) had significantly higher TG levels after NAC than those without (*P* = 0.049). Patients with a high ALC before NAC had significantly higher TG levels after NAC than patients with all breast cancer (*P* = 0.001), HER2-enriched breast cancer (*P* = 0.021), and TNBC (*P* = 0.008). Patients with a low NLR before NAC had significantly higher TG levels after NAC only among patients with TNBC (*P* = 0.025). In patients with human epidermal growth factor receptor 2-enriched breast cancer, the group with normal TC levels before NAC had significantly better OS than those with high TC levels (*P* = 0.013, log-rank test), and in patients with TNBC, the group with high TC levels after NAC had significantly better OS than those with normal TC levels (*P* = 0.014, log-rank test).

**Conclusions:**

Good systemic immune activity and chemosensitivity may be associated with lipid metabolism regulated by NAC in TNBC patients.

**Supplementary Information:**

The online version contains supplementary material available at 10.1186/s40001-022-00964-w.

## Background

Neoadjuvant chemotherapy (NAC) is the gold standard treatment for breast cancer and its use has increased the rate of breast-conserving surgery [1, 2]. In addition, the pathological complete response (pCR) after NAC is a predictor of good outcome, and its prognostic value is greatest in aggressive subtypes, human epidermal growth factor receptor 2 (HER2)-enriched, and triple-negative breast cancer (TNBC) [3]. These intrinsic breast cancer subtypes have a high malignancy and immune activity. We have reported previously that immune-related biomarkers, including the neutrophil–lymphocyte ratio (NLR) or tumor-infiltrating lymphocytes in biopsy specimens before NAC, are associated significantly with high pCR rates in these breast cancer subtypes [4].

Immunometabolism has become a relatively new field in cancer immunotherapy, and it has been recognized that the regulation of metabolism can enhance antitumor immunity [5–7]. In the case of lipid metabolism, obesity has been shown to be associated positively with breast cancer risk in postmenopausal hormone receptor-positive women [8, 9]. In addition, while epidemiological evidence has shown no association between the use of agents for dyslipidemia, mainly statins, and reduced breast cancer incidence, it supports a protective effect of these drugs on reducing breast cancer recurrence or mortality [10]. Moreover, several basic studies have shown that statins can suppress cancer cell proliferation, exert anti-angiogenic effects, and reduce the invasiveness and metastatic potential of breast cancer cells [11–16]. In addition, Crocetto et al. reported that the lipid alterations may be a potential tumor biomarker to detect bladder cancer, endocrine-related cancer, in clinical practice [17]; however, the relationship between lipid metabolism and immune activity in breast cancer patients has not been sufficiently investigated.

On the other hand, chemotherapy enhances antitumor immune responses [18, 19]. Some studies have revealed that changes in the lymphocytic subpopulations after NAC can be used as prognostic markers in patients with breast cancer patients [20–23]. In contrast, other studies have examined the metabolic changes before and after chemotherapy in breast cancer patients and showed significant changes in lipid levels [24, 25]. Furthermore, Tian et al*.* reported that NAC exerts an adverse effect on lipid levels during chemotherapy [26].

The present study investigated the correlation between lipid metabolism, antitumor immune responses, and chemosensitivity in patients with breast cancer treated with NAC.

## Methods

### Patient background

Data from the Osaka City University Graduate School of Medicine (Osaka, Japan) between April 2007 and March 2018 were analyzed. A total of 351 patients were diagnosed with early stage breast cancer (stage IIA, IIB, IIIA) and underwent with primary systemic treatment and curative surgery. We excluded 24 patients treated with neoadjuvant endocrine therapy and included 327 patients treated with NAC in this study. T and N factors and tumor stage were stratified based on the TNM Classification, UICC Seventh Edition [27]. Tumors were classified into intrinsic subtypes according to the immunohistochemical expression of the estrogen receptor (ER), progesterone receptor (PgR), and HER2. We defined ER + and/or PgR + and HER2-breast cancer as luminal, ER + and/or PgR + and HER2 + breast cancer as luminal-HER2, ER- and PgR-, HER2 + breast cancer as HER2-enriched, and ER-, PgR-, and HER2-breast cancer as TNBC. The antitumor effect was assessed according to the Response Evaluation Criteria in Solid Tumors [28]. The objective response (OR) was calculated as the sum of the clinical partial response and complete response (CR). All the patients underwent mastectomy or breast-conserving surgery after NAC. The pCR was defined as the complete disappearance of the invasive compartment of the lesion with or without intraductal components, including the lymph nodes” [29]. Postoperative adjuvant therapy suitable for each intrinsic breast cancer subtype was performed, and standard postoperative radiotherapy to the remnant breast was administered if necessary. All patients underwent physical examinations, blood tests, ultrasonography, computed tomography, and bone scintigraphy scans. Overall survival (OS) was defined as the time from curative surgery to death from any cause, and recurrence-free survival (RFS) was defined as freedom from all locoregional and distant recurrences. The median follow-up time for the assessment of OS was 5.5 years (range 0.2–12.4 years) and for RFS was 4.9 years (range 0.1–12.0 years).

### Blood sample analysis

Peripheral blood samples were obtained at the time of diagnosis, and preoperative blood samples were obtained within a week before surgery. We evaluated the serum lipid levels, including total cholesterol (TC) levels [categorized as low (≤ 149 mg/dl), normal (150–219 mg/dl), and high (≥ 220 mg/dl)] and triglyceride (TG) levels [categorized as low (≤ 49 mg/dl), normal (50–149 mg/dl), and high (≥ 150 mg/dl)]. The differential white blood cell counts were analyzed using a Coulter LH 750 Hematology Analyzer (Beckman Coulter, Brea, CA, USA). The neutrophil-to-lymphocyte ratio (NLR) was calculated from the blood samples by dividing the absolute neutrophil count by the absolute lymphocyte count (ALC).

### Statistical analyses

Statistical analyses were performed using the JMP13 software (SAS Institute, Cary, NC, USA). Associations among the variables were analyzed using the χ^2^ or Fisher’s exact test, as appropriate. OS and RFS were estimated using the Kaplan–Meier method and log-rank test. Statistical significance was set at *P* < 0.05.

## Results

### Clinicopathological responses of all the breast cancer patients to NAC

The differences in clinicopathological features due to intrinsic breast cancer subtypes are presented in Table 1. A total of 327 patients were included in this study. Among these, 108 (33.0%), 42 (12.9%), 72 (22.0%), and 105 (32.1%) had luminal, luminal-HER2, HER2-enriched, and TNBC, respectively. NAC-related pCR was observed in 121 patients (37.0%). The evaluation based on the clinicopathological features revealed that the pCR rate was significantly higher in HER2-enriched (59.7%, 43/72) and TNBC patients (44.8%, 47/105) (*P* < 0.001). OR was observed in 295 patients (90.2%). The OR rate was high in all the breast cancer subtypes, and no significant differences were observed (*P* = 0.070). Patients with high TC levels increased after NAC in each breast cancer subtypes other than HER2-enriched. Furthermore, patients with TG levels increased after NAC in all subtypes, and the rate of change was highest especially in TNBC (21.0% → 48.1%).

**Table 1:** Differences in clinicopathological features due to intrinsic breast cancer subtypes in 327 patients

Parameters	Intrinsic subtype				*P* value
	Luminal (*n* = 108)	Luminal-HER2 (*n* = 42)	HER2-enriched (*n* = 72)	TNBC (*n* = 105)	
*Age at operation*					0.054
≤ 54	59 (54.6%)	24 (57.1%)	26 (36.1%)	55 (52.4%)	
> 54	49 (45.4%)	18 (42.9%)	46 (63.9%)	50 (47.6%)	
*Menopause*					0.006
Pre-	47 (43.5%)	22 (52.4%)	16 (22.9%)	36 (35.0%)	
Post-	61 (56.5%)	20 (47.6%)	54 (77.1%)	67 (65.0%)	
*BMI*					0.341
≤ 22.0	48 (44.4%)	21 (50.0%)	42 (58.3%)	53 (50.5%)	
> 22.0	60 (55.6%)	21 (50.0%)	30 (41.7%)	52 (49.5%)	
*Tumor size*					0.528
≤ 2 cm	13 (12.0%)	9 (21.4%)	9 (12.5%)	14 (13.3%)	
> 2 cm	95 (88.0%)	33 (78.6%)	63 (87.5%)	91 (86.7%)	
*Lymph node status*					0.011
Negative	27 (25.0%)	22 (52.4%)	26 (36.1%)	30 (28.6%)	
Positive	81 (75.0%)	20 (47.6%)	46 (63.9%)	75 (71.4%)	
*TC (preNAC)*					0.632
Normal	52 (54.2%)	21 (60.0%)	39 (60.9%)	49 (51.6%)	
High	44 (45.8%)	14 (40.0%)	25 (39.1%)	46 (48.4%)	
*TG (preNAC)*					0.999
Normal	76 (79.2%)	28 (77.8%)	48 (78.7%)	75 (79.0%)	
High	20 (20.8%)	8 (22.2%)	13 (21.3%)	20 (21.0%)	
*TC (postNAC)*					0.014
Normal	35 (37.6%)	15 (50.0%)	36 (62.1%)	33 (38.4%)	
High	58 (62.4%)	15 (50.0%)	22 (37.9%)	53 (61.6%)	
*TG (postNAC)*					0.649
Normal	53 (58.9%)	16 (55.2%)	33 (62.3%)	42 (51.9%)	
High	37 (41.1%)	13 (44.8%)	20 (37.7%)	39 (48.1%)	
*Objective response rate*					0.070
Non-ORR	11 (10.2%)	6 (14.3%)	2 (2.8%)	13 (12.4%)	
ORR	97 (89.8%)	36 (85.7%)	70 (97.2%)	92 (87.6%)	
*Pathological response*					< 0.001
Non-pCR	89 (82.4%)	30 (71.4%)	29 (40.3%)	58 (55.2%)	
pCR	19 (17.6%)	12 (28.6%)	43 (59.7%)	47 (44.8%)	

*BMI*, body mass index; *HER2*, human epidermal growth factor receptor 2; *NAC*, neoadjuvant chemotherapy; *ORR*, objective response rate; *pCR*, pathological complete response; *TC*, total-cholesterol; *TG*, triglyceride; *TNBC*, triple-negative breast cancer

In all breast cancer patients, RFS and OS were significantly longer in patients who achieved pCR than in those who did not (*P* < 0.001 and *P* = 0.006, log-rank, respectively; Additional file 1: Fig. S1a, Additional file 2: Fig. S2a). Furthermore, these outcomes were also significantly better in patients who achieved OR than in those who did not (*P* < 0.001 and *P* = 0.001, log-rank, respectively; Additional file 3: Figs. S3a, Additional file 4: Fig. S4a). In addition, we investigated the prognostic factors for RFS and OS for each breast cancer subtype. Among patients with luminal cancer, no significant differences were observed in RFS (*P* = 0.882, *P* = 0.399, log-rank, respectively) and OS (*P* = 0.861, *P* = 0.202, log-rank, respectively) according to the clinicopathological responses, pCR, and OR (Additional file 1: Fig. S1b, Additional file 2: Fig. S2b, Additional file 3: Fig. S3b, Additional file 4: Fig. S4b). In contrast, among the patients with TNBC, RFS (*P* = 0.005, *P* < 0.001, log-rank, respectively) and OS (*P* = 0.003, *P* < 0.001, log-rank, respectively) were significantly longer in patients who achieved pCR or OR than in those who did not (Additional file 1: Fig. S1e, Additional file 2: Fig. S2e, Additional file 3: Fig. S3e, Additional file 4: Fig. S4e).

**Fig. 1:**
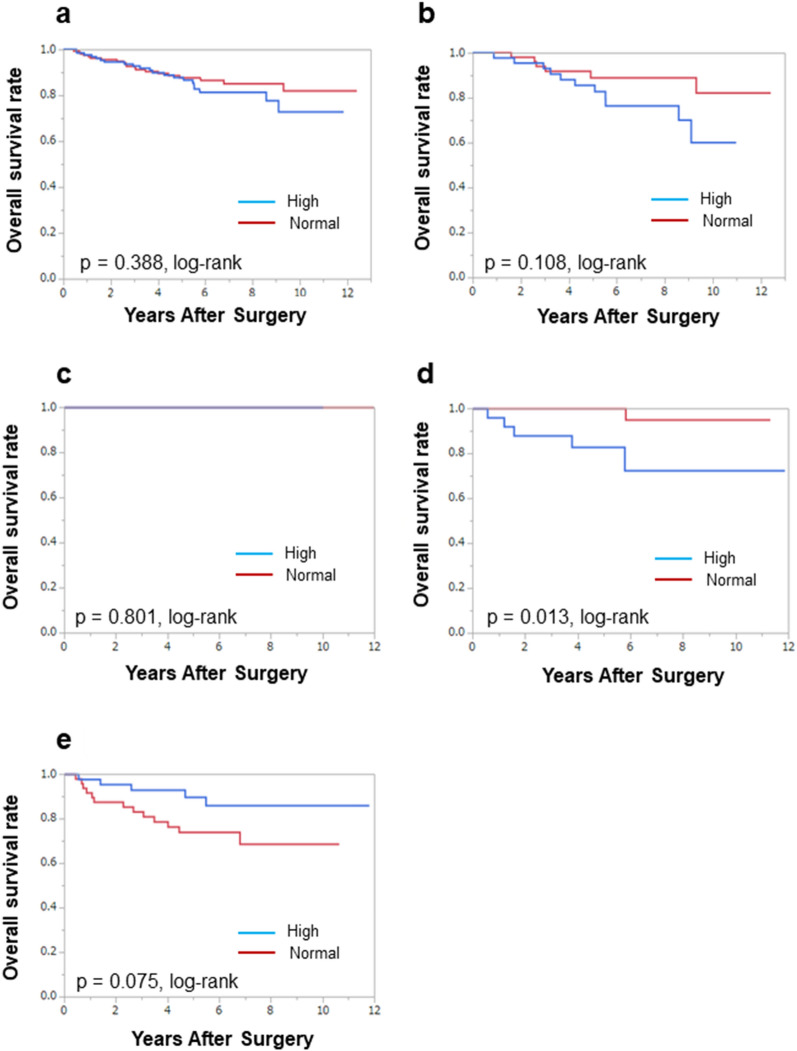
Analysis of total cholesterol (TC) before NAC and overall survival (OS) in patients with all breast cancer subtypes. OS was not significantly different between patients with normal TC and high TC levels among all breast cancer (*P* = 0.388, log-rank) (**a**). OS was not significantly different between patients with normal TC and high TC levels in luminal breast cancer (*P* = 0.108, log-rank) (**b**). OS was not significantly different between patients with normal TC and high TC levels in luminal-human-epidermal growth factor receptor 2 (HER2)-enriched breast cancer (*P* = 0.801, log-rank) (**c**). Patients with normal TC before NAC had significantly better OS of HER2-enriched (*P* = 0.013, log-rank) (**d**). OS was not significantly different between patients with normal TC and high TC levels in triple-negative breast cancer (*P* = 0.075, log-rank) (**e**)

### Analysis of relationships between lipid metabolism and chemosensitivity and prognosis

The relationship between lipid metabolism and chemosensitivity was examined (Table 2). There were no significant correlations between lipid metabolism and the pCR in any breast cancer subtype. In contrast, only TNBC patients with OR had significantly higher TG levels after NAC than patients without OR (*P* = 0.049).

**Table 2:** Relationships between lipid metabolism and chemosensitivity

	pCR			OR		
	Negative	Positive	*P* value	Negative	Positive	*P* value
*All breast cancer (n = 327)*						
TC (preNAC)			0.626			0.242
Normal	98 (54.1%)	63 (57.8%)		13 (44.8%)	148 (56.7%)	
High	83 (45.9%)	46 (42.2%)		16 (55.2%)	113 (43.3%)	
TG (preNAC)			0.299			0.866
Normal	139 (76.8%)	88 (82.2%)		24 (80.0%)	203 (78.7%)	
High	42 (23.2%)	19 (17.8%)		6 (20.0%)	55 (21.3%)	
TC (postNAC)			0.703			0.952
Normal	72 (43.4%)	47 (46.5%)		11 (44.0%)	108 (44.6%)	
High	94 (56.6%)	54 (53.5%)		14 (56.0%)	134 (55.4%)	
TG (postNAC)			0.694			0.667
Normal	88 (55.7%)	56 (59.0%)		15 (62.5%)	129 (56.3%)	
High	70 (44.3%)	39 (41.0%)		9 (37.5%)	100 (43.7%)	
*Luminal (n = 108)*						
TC (preNAC)			0.795			0.728
Normal	43 (55.1%)	9 (50.0%)		4 (44.4%)	48 (55.2%)	
High	35 (44.9%)	9 (50.0%)		5 (55.6%)	39 (44.8%)	
TG (preNAC)			0.347			0.680
Normal	60 (76.9%)	16 (88.9%)		8 (88.9%)	68 (78.2%)	
High	18 (23.1%)	2 (11.1%)		1 (11.1%)	19 (21.8%)	
TC (postNAC)			0.415			0.707
Normal	27 (35.5%)	8 (47.1%)		2 (28.6%)	33 (38.4%)	
High	49 (64.5%)	9 (52.9%)		5 (71.4%)	53 (61.6%)	
TG (postNAC)			0.785			0.685
Normal	42 (57.5%)	11 (64.7%)		4 (66.7%)	49 (58.3%)	
High	31 (42.5%)	6 (35.3%)		2 (33.3%)	35 (41.7%)	
*Luminal-HER (n = 42)*						
TC (preNAC)			0.766			1.000
Normal	14 (58.3%)	7 (63.6%)		3 (60.0%)	18 (60.0%)	
High	10 (41.7%)	4 (36.4%)		2 (40.0%)	12 (40.0%)	
TG (preNAC)			0.076			0.109
Normal	17 (68.0%)	11 (100.0%)		3 (50.0%)	25 (83.3%)	
High	8 (32.0%)	0 (0.0%)		3 (50.0%)	5 (16.7%)	
TC (postNAC)			1.000			0.330
Normal	10 (50.0%)	5 (50.0%)		1 (20.0%)	14 (56.0%)	
High	10 (50.0%)	5 (50.0%)		4 (80.0%)	11 (44.0%)	
TG (postNAC)			0.130			0.144
Normal	9 (45.0%)	7 (77.8%)		1 (20.0%)	15 (62.5%)	
High	11 (55.0%)	2 (22.2%)		4 (80.0%)	9 (37.5%)	
*HER2-enriched (n = 72)*						
TC (preNAC)			0.436			0.750
Normal	14 (53.9%)	25 (65.8%)		1 (50.0%)	38 (61.3%)	
High	12 (46.1%)	13 (34.2%)		1 (50.0%)	24 (38.7%)	
TG (preNAC)			0.835			0.323
Normal	20 (80.0%)	28 (77.8%)		2 (100.0%)	46 (78.0%)	
High	5 (20.0%)	8 (22.2%)		0 (0.0%)	13 (22.0%)	
TC (postNAC)			0.792			0.521
Normal	15 (60.0%)	21 (63.6%)		2 (100.0%)	34 (60.7%)	
High	10 (40.0%)	12 (36.4%)		0 (0.0%)	22 (39.3%)	
TG (postNAC)			0.779			0.719
Normal	15 (65.2%)	18 (60.0%)		1 (50.0%)	32 (62.8%)	
High	8 (34.8%)	12 (40.0%)		1 (50.0%)	19 (37.2%)	
*TNBC (n = 105)*						
TC (preNAC)			0.889			0.378
Normal	27 (50.9%)	22 (52.4%)		5 (38.5%)	44 (53.7%)	
High	26 (49.1%)	20 (47.6%)		8 (61.5%)	38 (46.3%)	
TG (preNAC)			0.936			0.729
Normal	42 (79.3%)	33 (78.6%)		11 (84.6%)	64 (78.1%)	
High	11 (20.7%)	9 (21.4%)		2 (15.4%)	18 (21.9%)	
TC (postNAC)			0.270			0.322
Normal	20 (44.5%)	13 (31.7%)		6 (54.6%)	27 (36.0%)	
High	25 (55.6%)	28 (68.3%)		5 (45.4%)	48 (64.0%)	
TG (postNAC)			0.921			0.049
Normal	22 (52.4%)	20 (51.3%)		9 (81.8%)	33 (47.1%)	
High	20 (47.6%)	19 (48.7%)		2 (18.2%)	37 (52.9%)	

*HER2*, human epidermal growth factor receptor 2; *NAC*, neoadjuvant chemotherapy; *TC*, total-cholesterol; *TG*, triglyceride; *TNBC*, triple-negative breast cancer

**Fig. 2:**
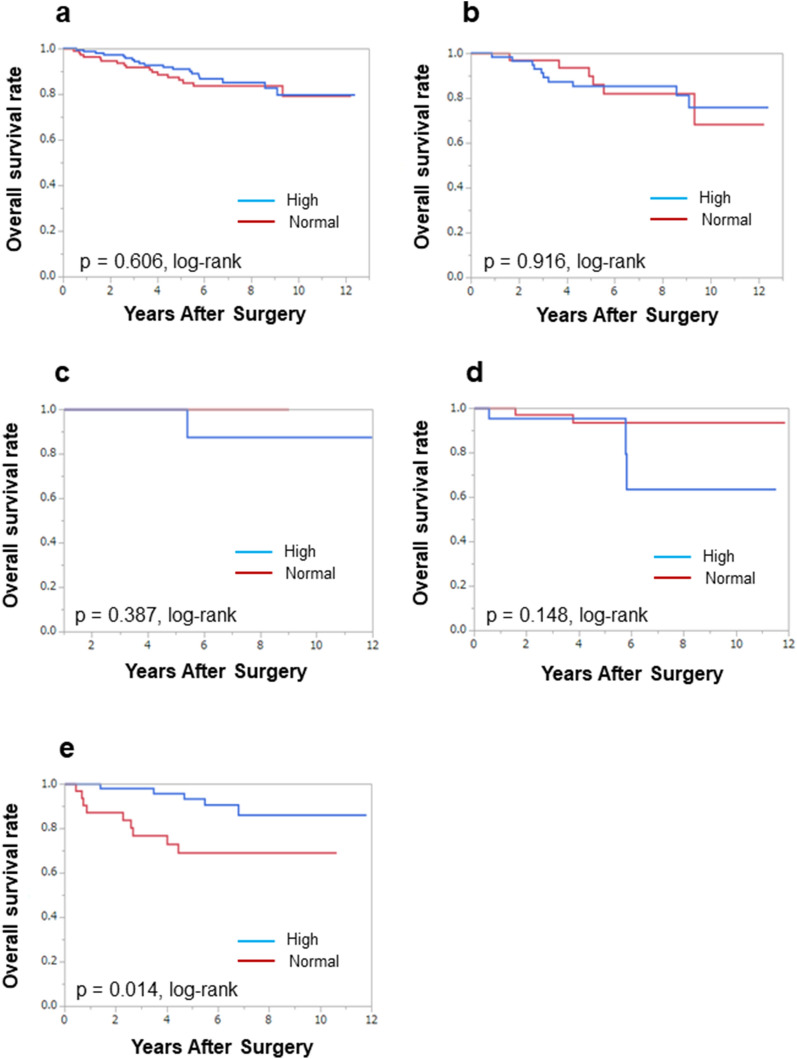
Analysis of total cholesterol (TC) after NAC and overall survival (OS) in patients with all breast cancer subtypes. OS was not significantly different between patients with normal TC and high TC levels among all breast cancer (*P* = 0.606, log-rank) (**a**). OS was not significantly different between patients with normal TC and high TC levels in luminal breast cancer (*P* = 0.916, log-rank) (**b**). OS was not significantly different between patients with normal TC and high TC levels in luminal-human-epidermal growth factor receptor 2 (HER2)-enriched breast cancer (*P* = 0.387, log-rank) (**c**). OS was not significantly different between patients with normal TC and high TC levels in HER2-enriched breast cancer (*P* = 0.148, log-rank) (**d**). Patients with high TC after NAC had significantly better OS of triple-negative breast cancer (*P* = 0.014, log-rank) (**e**)

We also investigated the prognostic value of serum lipid levels before and after NAC for each intrinsic breast cancer subtype. In patients with HER2-enriched breast cancer, those with normal TC levels before NAC had a significantly better OS than those with high TC levels (*P* = 0.013, log-rank test) (Fig. 1d), and in patients with TNBC, the group with high TC levels after NAC had significantly better OS than those with normal TC levels (*P* = 0.014, log-rank test) (Fig. 2e). There was no association between recurrence and TC levels. Also, there was no relationship between the prognosis and triglyceride levels before and after NAC (Additional files 5–10: Figs. S5–S10).

### Analysis of relationships between lipid metabolism and immune activity

The pre-NAC ALC ranged from 712.8 to 4446.2 (mean, 1811.0; median, 1749; standard deviation, 613.9), and the pre-NAC NLR ranged from 0.5 to 10.6 (mean, 2.3; median, 2.0; standard deviation, 1.2). The post-NAC ALC ranged from 285.6 to 3697.7 (mean, 1122.4; median, 1005.4; standard deviation, 517.0), and the post-NAC NLR ranged from 0.3 to 15.9 (mean, 2.9; median, 2.4; standard deviation, 1.9). We defined the pre-NAC median as the cutoff value for the ALC and NLR. There were no significant correlations between the systemic immune activity and the effect of NAC in all the breast cancer patients or each of the breast cancer subtypes (Additional file 11: Table S1).

The relationship between lipid metabolism and systemic immune activity is shown in Table 3. Patients with a high ALC before NAC had significantly higher TG levels after NAC in all the breast cancers (*P* = 0.001). In addition, among the patients with HER2-enriched breast cancer, high TG levels after NAC were associated significantly with a high ALC before NAC (*P* = 0.021), and high TG levels before NAC were associated significantly with a high ALC after NAC (*P* = 0.046). Furthermore, among patients with TNBC, high TG levels after NAC were associated significantly with a high ALC (*P* = 0.008) and a low NLR (*P* = 0.025) before NAC, while high TG levels before NAC were associated significantly with a low NLR after NAC (*P* = 0.034).

**Table 3:** Relationships between lipid metabolism and immune activity

	ALC (pre-NAC)			NLR (pre-NAC)			ALC (post-NAC)			NLR (post-NAC)		
	Low	High	*P* value	Low	High	*P* value	Low	High	*P* value	Low	High	*P* value
*All breast cancer (n = 327)*												
TC (preNAC)			0.638			0.479			0.564			0.978
Normal	82 (56.9%)	79 (54.1%)		76 (53.2%)	85 (57.8%)		145 (56.2%)	15 (50.0%)		59 (55.7%)	101 (55.5%)	
High	62 (43.1%)	67 (45.9%)		67 (46.8%)	62 (42.2%)		113 (43.8%)	15 (50.0%)		47 (44.3%)	81 (44.5%)	
TG (preNAC)			0.249			0.886			0.101			0.655
Normal	117 (81.8%)	110 (75.9%)		112 (79.4%)	115 (78.2%)		205 (80.1%)	20 (66.7%)		81 (77.1%)	144 (79.6%)	
High	26 (18.2%)	35 (24.1%)		29 (20.6%)	32 (21.8%)		51 (19.9%)	10 (33.3%)		24 (22.9%)	37 (20.4%)	
TC (postNAC)			0.388			0.219			0.292			0.163
Normal	60 (47.6%)	59 (41.8%)		60 (41.1%)	59 (48.8%)		105 (43.8%)	14 (56.0%)		51 (50.5%)	68 (41.5%)	
High	66 (52.4%)	82 (58.2%)		86 (58.9%)	62 (51.2%)		135 (56.2%)	11 (44.0%)		50 (49.5%)	96 (58.5%)	
TG (postNAC)			0.001			0.164			0.803			0.974
Normal	81 (68.6%)	63 (46.7%)		73 (52.9%)	71 (61.7%)		129 (56.8%)	13 (54.2%)		55 (56.7%)	87 (56.5%)	
High	37 (31.4%)	72 (53.3%)		65 (47.1%)	44 (38.3%)		98 (43.2%)	11 (45.8%)		42 (43.3%)	67 (43.5%)	
*Luminal (n = 108)*												
TC (preNAC)			0.54			0.838			0.728			0.665
Normal	25 (58.1%)	27 (50.9%)		28 (52.8%)	24 (55.8%)		48 (55.2%)	4 (44.4%)		16 (50.0%)	36 (56.3%)	
High	18 (41.9%)	26 (49.1%)		25 (47.2%)	19 (44.2%)		39 (44.8%)	5 (55.6%)		16 (50.0%)	28 (43.7%)	
TG (preNAC)			0.801			0.801			0.915			0.064
Normal	35 (81.4%)	41 (77.4%)		41 (77.4%)	35 (81.4%)		69 (79.3%)	7 (77.8%)		29 (90.6%)	47 (73.4%)	
High	8 (18.6%)	12 (22.6%)		12 (22.6%)	8 (18.6%)		18 (20.7%)	2 (22.2%)		3 (9.4%)	17 (26.6%)	
TC (postNAC)			0.853			0.667			0.926			0.38
Normal	15 (36.6%)	20 (38.5%)		20 (35.7%)	15 (40.5%)		31 (37.8%)	4 (36.4%)		16 (44.4%)	19 (33.3%)	
High	26 (63.4%)	32 (61.5%)		36 (64.3%)	22 (59.5%)		51 (62.2%)	7 (63.6%)		20 (55.6%)	38 (66.7%)	
TG (postNAC)			0.397			0.386			0.515			0.661
Normal	25 (64.1%)	28 (54.9%)		34 (63.0%)	19 (52.8%)		45 (57.0%)	8 (72.7%)		22 (62.9%)	31 (56.4%)	
High	14 (35.9%)	23 (45.1%)		20 (37.0%)	17 (47.2%)		34 (43.0%)	3 (27.3%)		13 (37.1%)	24 (43.6%)	
*Luminal-HER (n = 42)*												
TC (preNAC)			0.176			0.491			0.135			0.774
Normal	13 (72.2%)	8 (47.1%)		10 (52.6%)	11 (68.8%)		19 (65.5%)	1 (20.0%)		9 (56.3%)	11 (61.1%)	
High	5 (27.8%)	9 (52.9%)		9 (47.4%)	5 (31.2%)		10 (34.5%)	4 (80.0%)		7 (43.7%)	7 (38.9%)	
TG (preNAC)			0.695			0.114			0.868			0.7
Normal	14 (73.7%)	14 (82.4%)		17 (89.5%)	11 (64.7%)		23 (76.7%)	4 (80.0%)		13 (81.3%)	14 (73.7%)	
High	5 (26.3%)	3 (17.6%)		2 (10.5%)	6 (35.3%)		7 (23.3%)	1 (20.0%)		3 (18.7%)	5 (26.3%)	
TC (postNAC)			0.462			0.715			0.96			0.837
Normal	10 (58.8%)	5 (38.5%)		8 (57.1%)	7 (43.8%)		14 (51.9%)	1 (50.0%)		7 (53.9%)	8 (50.0%)	
High	7 (41.2%)	8 (61.5%)		6 (42.9%)	9 (56.2%)		13 (48.1%)	1 (50.0%)		6 (46.1%)	8 (50.0%)	
TG (postNAC)			0.274			0.897			0.206			0.705
Normal	11 (64.7%)	5 (41.7%)		7 (53.9%)	9 (56.3%)		15 (57.7%)	0 (0.00%)		6 (46.2%)	9 (60.0%)	
High	6 (35.3%)	7 (58.3%)		6 (46.1%)	7 (43.7%)		11 (42.3%)	2 (100.0%)		7 (53.8%)	6 (40.0%)	
*HER2-enriched (n = 72)*												
TC (preNAC)			0.955			0.935			0.738			0.935
Normal	20 (60.6%)	19 (61.3%)		16 (61.5%)	23 (60.5%)		33 (62.3%)	6 (54.6%)		16 (61.5%)	23 (60.5%)	
High	13 (39.4%)	12 (38.7%)		10 (38.5%)	15 (39.5%)		20 (37.7%)	5 (45.4%)		10 (38.5%)	15 (39.5%)	
TG (preNAC)			0.363			0.539			0.046			0.349
Normal	26 (83.9%)	22 (73.3%)		20 (83.3%)	28 (75.7%)		42 (84.0%)	6 (54.6%)		18 (72.0%)	30 (83.3%)	
High	5 (16.1%)	8 (26.7%)		4 (16.7%)	9 (24.3%)		8 (16.0%)	5 (45.4%)		7 (28.0%)	6 (16.7%)	
TC (postNAC)			0.593			0.787			0.697			0.267
Normal	16 (66.7%)	20 (58.8%)		17 (58.6%)	19 (65.5%)		30 (60.0%)	6 (75.0%)		16 (72.7%)	20 (55.6%)	
High	8 (33.3%)	14 (41.2%)		12 (41.4%)	10 (34.5%)		20 (40.0%)	2 (25.0%)		6 (27.3%)	16 (44.4%)	
TG (postNAC)			0.021			0.264			0.766			0.779
Normal	18 (81.8%)	15 (48.4%)		14 (53.9%)	19 (70.4%)		29 (63.0%)	4 (57.1%)		13 (65.0%)	20 (60.6%)	
High	4 (18.2%)	16 (51.6%)		12 (46.1%)	8 (29.6%)		17 (37.0%)	3 (42.9%)		7 (35.0%)	13 (39.4%)	
*TNBC (n = 105)*												
TC (preNAC)			0.539			0.683			0.364			0.664
Normal	24 (48.0%)	25 (55.6%)		22 (48.9%)	27 (54.0%)		45 (50.6%)	4 (80.0%)		18 (56.3%)	31 (50.0%)	
High	26 (52.0%)	20 (44.4%)		23 (51.1%)	23 (46.0%)		44 (49.4%)	1 (20.0%)		14 (43.7%)	31 (50.0%)	
TG (preNAC)			0.22			0.462			0.287			0.034
Normal	42 (84.0%)	33 (73.3%)		34 (75.6%)	41 (82.0%)		71 (79.8%)	3 (60.0%)		21 (65.6%)	53 (85.5%)	
High	8 (16.0%)	12 (26.7%)		11 (24.4%)	9 (18.0%)		18 (20.2%)	2 (40.0%)		11 (34.4%)	9 (14.5%)	
TC (postNAC)			0.382			0.19			0.294			0.87
Normal	19 (43.2%)	14 (33.3%)		15 (31.9%)	18 (46.2%)		30 (37.0%)	3 (75.0%)		12 (40.0%)	21 (38.2%)	
High	25 (56.8%)	28 (66.7%)		32 (68.1%)	21 (53.8%)		51 (63.0%)	1 (25.0%)		18 (60.0%)	34 (61.8%)	
TG (postNAC)			0.008			0.025			0.353			0.817
Normal	27 (67.5%)	15 (36.6%)		18 (40.0%)	24 (66.7%)		40 (52.6%)	1 (25.0%)		14 (48.3%)	27 (52.9%)	
High	13 (32.5%)	26 (63.4%)		27 (60.0%)	12 (33.3%)		36 (47.4%)	3 (75.0%)		15 (51.7%)	24 (47.1%)	

*ALC*, absolute lymphocyte count; *HER2*, human epidermal growth factor receptor 2; *NAC*, neoadjuvant chemotherapy; *NLR*, neutrophil-to-lymphocyte ratio; *TC*, total-cholesterol; *TG*, triglyceride; *TNBC*, triple-negative breast cancer

## Discussion

In the present study, NAC increased serum TG levels, particularly in patients with TNBC. Some previous studies showed that serum lipid levels increased significantly after chemotherapy and that the TG levels may be a sensitive biomarker for determining the effect of adjuvant chemotherapy [24, 30]. Many anticancer drugs are metabolized in liver and may cause non-alcoholic fatty liver disease by variety of mechanisms [31]. However, this phenomenon has not yet been fully studied. To the best of our knowledge, our study was the first to analyze the predictive value of lipid metabolism for chemosensitivity of breast cancer patients treated with NAC and to stratify the intrinsic subtypes of breast cancer.

In this study, patients with reduced tumor size had significantly higher TG levels after NAC in only TNBC. Sharma et al*.* reported that some chemotherapy agents affect serum lipid levels by regulating the expression of genes involved in lipid metabolism in liver cells [32]. Therefore, it is considered that there is a correlation between the effects of NAC and lipid metabolism in TNBC.

The efficacy of NAC, especially in terms of the pCR, is currently acknowledged as an indicator of good outcomes in patients with TNBC and HER2-enriched breast cancer, which have high immune activity [3, 33, 34]. Hence, it is expected that there will be an association between lipid metabolism and tumor immune activity in TNBC. Recent studies have reported that the regulation of metabolism can affect the tumor immune microenvironment and enhance the antitumor immune response [5–7]. In our study, good systemic immune activity, a high ALC, or low NLR before NAC were associated significantly with high TG levels after NAC in patients with TNBC or HER2-enriched breast cancer.

However, no relationships were observed between the pre-NAC lipid levels and NAC efficacy. In addition, the serum lipid levels before NAC showed no significant relationships with the ALC or NLR. Hence, it was difficult to predict chemosensitivity or systemic immune activity based on serum lipid levels prior to NAC.

In the present study, no significant associations were observed between the systemic immune activity and the effect of NAC. However, in our previous study, we set the cutoff value of pre-NAC NLR to 3.0, in the same breast cancer patients, and the pCR rate was significantly higher in TNBC patients with a good immune status, low NLR group [4]. This result suggested that not only the effect of tumor reduction, but also the effect of increasing serum lipid levels is recognized in patients with good systemic immune activity.

Although the TG levels after NAC may be an indicator of chemosensitivity in TNBC, they are not useful predictive markers of recurrence. The reason for this may be that changes in the lipid profiles after NAC are temporary [26]. We presumed that a favorable prognosis may not be based on lipid levels at the time of diagnosis or after NAC, but is induced by the maintenance good lipid metabolism after surgery.

This study has some limitations. First, this was a single-center, retrospective study, then the sample size was relatively small. Second, in our study, serum TC levels were associated with better OS in patients with HER2-enriched breast cancer or TNBC. However, we did not have detailed data on high-density lipoprotein cholesterol and low-density lipoprotein cholesterol levels. In addition, many factors influence serum lipid levels, including lifestyle and adherence to medication. Considering these limitations, further prospective multicenter studies are needed.

## Conclusions

This is the first study to demonstrate the clinical relationships between lipid metabolism, chemosensitivity, and systemic immune activity in patients with breast cancer treated with NAC. The findings of this study indicated that a good systemic immune activity and the effect of NAC may be associated with lipid metabolism regulated by chemotherapy in patients with TNBC.

## Supplementary Information

**Additional file 1**: **Fig. S1** Recurrence-free survival (RFS) using Kaplan–Meier method in patients based on pCR or non-pCR with different intrinsic breast cancer subtype. All breast cancer (**a**), Luminal (**b**), Luminal-human epidermal growth factor receptor 2 (HER2) (**c**), HER2-enrich (**d**) and triple-negative breast cancer (**e**).

**Additional file 2**: **Fig. S2.** Overall survival (OS) using Kaplan–Meier method in patients based on pCR or non-pCR with different intrinsic breast cancer subtype. All breast cancer (**a**), Luminal (**b**), Luminal-human epidermal growth factor receptor 2 (HER2) (**c**), HER2-enrich (**d**) and triple-negative breast cancer (**e**).

**Additional file 3**: **Fig. S3** Recurrence-free survival (RFS) using Kaplan–Meier method in patients based on OR or non-OR with different intrinsic breast cancer subtype. All breast cancer (**a**), Luminal (**b**), Luminal-human epidermal growth factor receptor 2 (HER2) (**c**), HER2-enrich (**d**) and triple-negative breast cancer (**e**).

**Additional file 4**: **Fig. S4** Overall survival (OS) using Kaplan–Meier method in patients based on OR or non-OR with different intrinsic breast cancer subtype. All breast cancer (**a**), Luminal (**b**), Luminal-human epidermal growth factor receptor 2 (HER2) (**c**), HER2-enrich (**d**) and triple-negative breast cancer (**e**).

**Additional file 5**: **Fig. S5** Recurrence-free survival (RFS) using Kaplan–Meier method in patients based on normal or high-total cholesterol before NAC with different intrinsic breast cancer subtype. All breast cancer (**a**), Luminal (**b**), Luminal-human epidermal growth factor receptor 2 (HER2) (**c**), HER2-enrich (**d**) and triple-negative breast cancer (**e**).

**Additional file 6**: **Fig. S6** Recurrence-free survival (RFS) using Kaplan–Meier method in patients based on normal or high-total cholesterol after NAC with different intrinsic breast cancer subtype. All breast cancer (**a**), Luminal (**b**), Luminal-human epidermal growth factor receptor 2 (HER2) (**c**), HER2-enrich (**d**) and triple-negative breast cancer (**e**).

**Additional file 7**: **Fig. S7** Recurrence-free survival (RFS) using Kaplan–Meier method in patients based on normal or high-triglyceride before NAC with different intrinsic breast cancer subtype. All breast cancer (**a**), Luminal (**b**), Luminal-human epidermal growth factor receptor 2 (HER2) (**c**), HER2-enrich (**d**) and triple-negative breast cancer (**e**).

**Additional file 8**: **Fig. S8** Overall survival (OS) using Kaplan–Meier method in patients based on normal or high-triglyceride before NAC with different intrinsic breast cancer subtype. All breast cancer (**a**), Luminal (**b**), Luminal-human epidermal growth factor receptor 2 (HER2) (**c**), HER2-enrich (**d**) and triple-negative breast cancer (**e**).

**Additional file 9**: **Fig. S9** Recurrence-free survival (RFS) using Kaplan–Meier method in patients based on normal or high-triglyceride after NAC with different intrinsic breast cancer subtype. All breast cancer (**a**), Luminal (**b**), Luminal-human epidermal growth factor receptor 2 (HER2) (**c**), HER2-enrich (**d**) and triple-negative breast cancer (**e**).

**Additional file 10**: **Fig. S10** Overall survival (RFS) using Kaplan–Meier method in patients based on normal or high-triglyceride after NAC with different intrinsic breast cancer subtype. All breast cancer (**a**), Luminal (**b**), Luminal-human epidermal growth factor receptor 2 (HER2) (**c**), HER2-enrich (**d**) and triple-negative breast cancer (**e**).** Table S1.** Relationships between immune activity and chemosensitivity.

**Additional file 11**: **Table S1.** Relationships between immune activity and chemosensitivity.

## Data Availability

The data sets used and/or analyzed during the current study are available from the corresponding author on reasonable request.

## Abbreviations

ALC: Absolute lymphocyte count
BMI: Body mass index
CR: Complete response
ER: Estrogen receptor
HER2: Human-epidermal growth factor receptor 2
NAC: Neoadjuvant chemotherapy
NLR: Neutrophil-to-lymphocyte ratio
OR: Objective response
ORR: Objective response rate
OS: Overall survival
pCR: Pathological complete response
PgR: Progesterone receptor
RFS: Recurrence-free survival
TC: Total-cholesterol
TG: Triglyceride
TNBC: Triple-negative breast cancer
