# Supplementary figures and images for: Clinical verification of the relationship between serum lipid metabolism and immune activity in breast cancer patients treated with neoadjuvant chemotherapy
Source: Eur J Med Res. 2023 Jan 2;28:2. doi: 10.1186/s40001-022-00964-w (PMC9806883; doi:10.1186/s40001-022-00964-w)

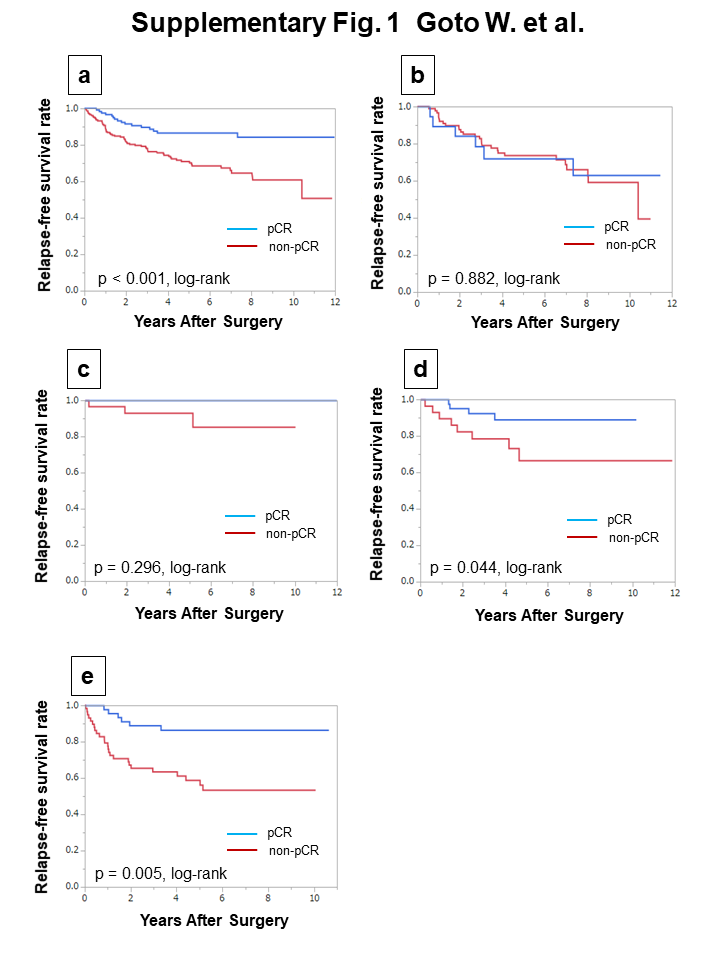

Supplement: Supplementary file 1 — Additional file 1: Fig. S1 Recurrence-free survival (RFS) using Kaplan–Meier method in patients based on pCR or non-pCR with different intrinsic breast cancer subtype. All breast cancer (a), Luminal (b), Luminal-human epidermal growth factor receptor 2 (HER2) (c), HER2-enrich (d) and triple-negative breast cancer (e). [file 40001_2022_964_MOESM1_ESM.tif]

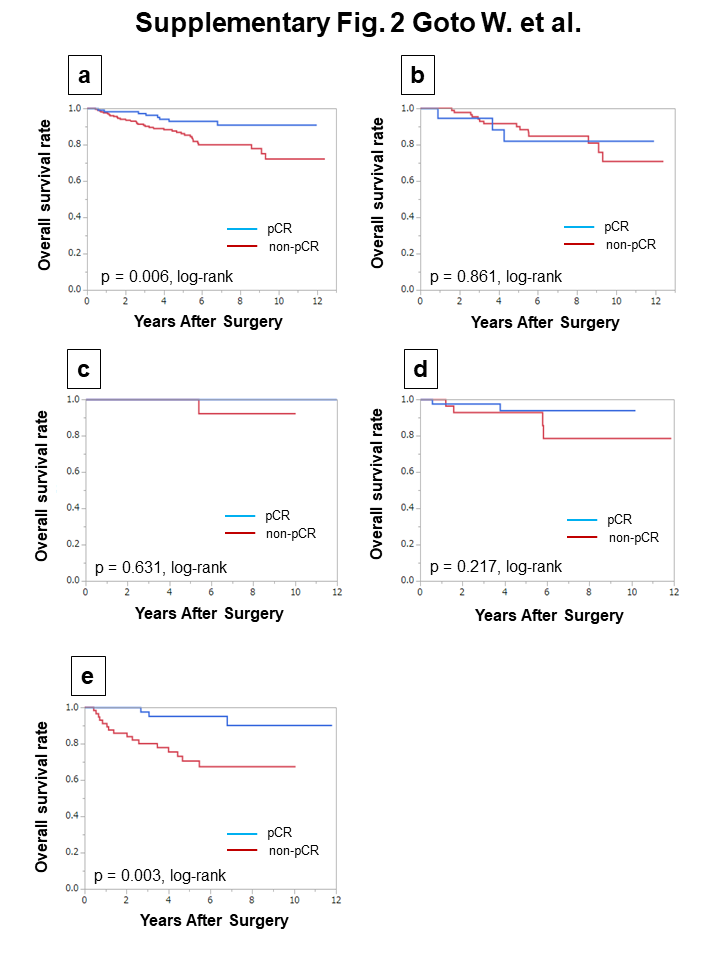

Supplement: Supplementary file 2 — Additional file 2: Fig. S2. Overall survival (OS) using Kaplan–Meier method in patients based on pCR or non-pCR with different intrinsic breast cancer subtype. All breast cancer (a), Luminal (b), Luminal-human epidermal growth factor receptor 2 (HER2) (c), HER2-enrich (d) and triple-negative breast cancer (e). [file 40001_2022_964_MOESM2_ESM.tif]

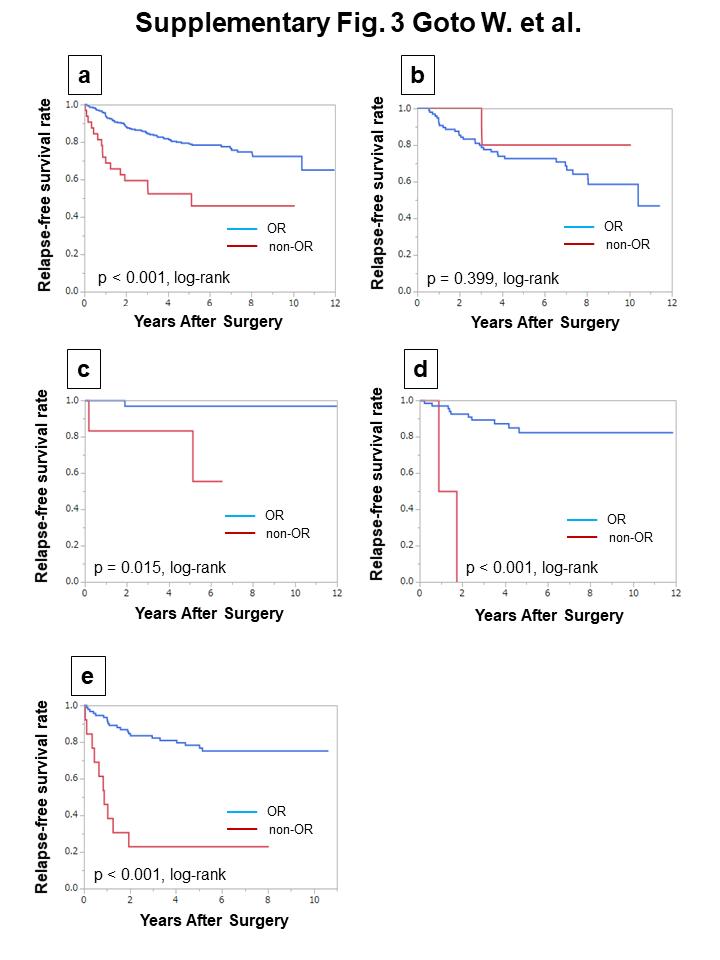

Supplement: Supplementary file 3 — Additional file 3: Fig. S3 Recurrence-free survival (RFS) using Kaplan–Meier method in patients based on OR or non-OR with different intrinsic breast cancer subtype. All breast cancer (a), Luminal (b), Luminal-human epidermal growth factor receptor 2 (HER2) (c), HER2-enrich (d) and triple-negative breast cancer (e). [file 40001_2022_964_MOESM3_ESM.tif]

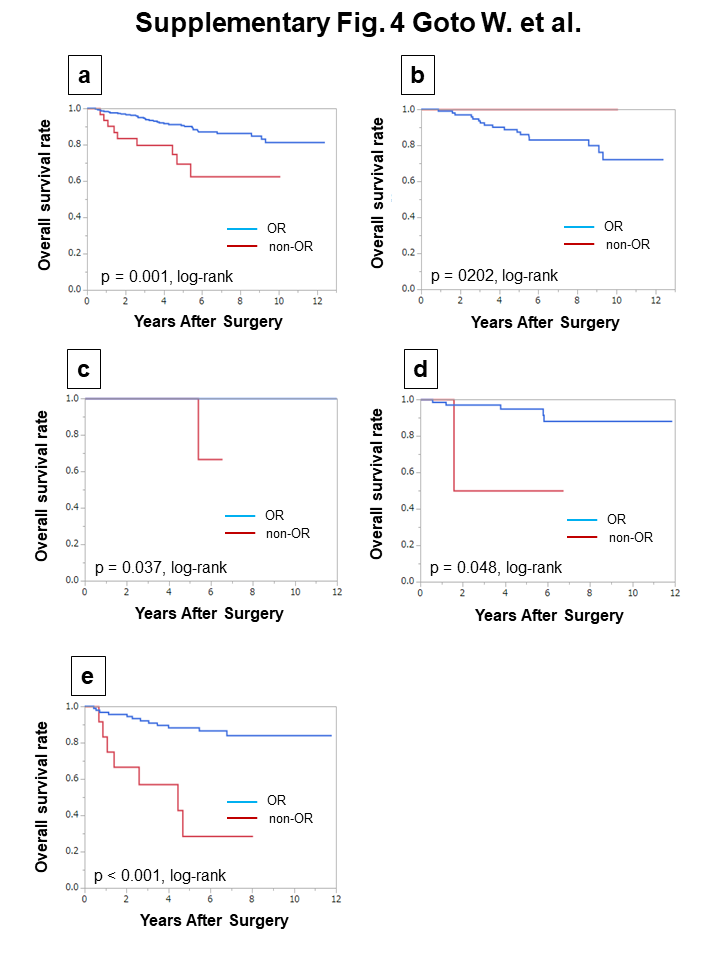

Supplement: Supplementary file 4 — Additional file 4: Fig. S4 Overall survival (OS) using Kaplan–Meier method in patients based on OR or non-OR with different intrinsic breast cancer subtype. All breast cancer (a), Luminal (b), Luminal-human epidermal growth factor receptor 2 (HER2) (c), HER2-enrich (d) and triple-negative breast cancer (e). [file 40001_2022_964_MOESM4_ESM.tif]

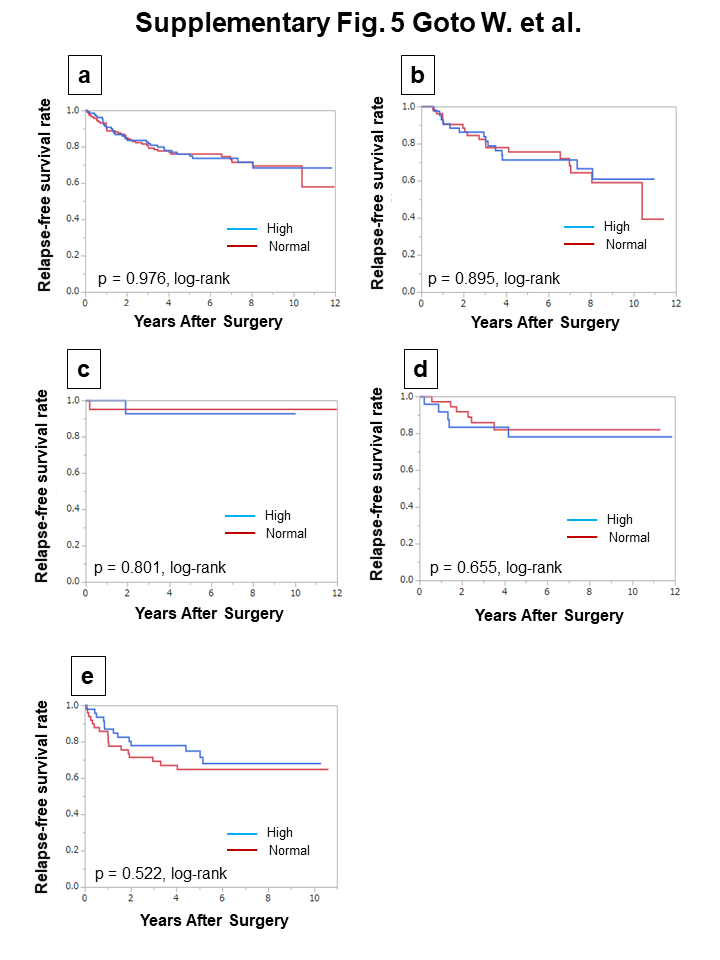

Supplement: Supplementary file 5 — Additional file 5: Fig. S5 Recurrence-free survival (RFS) using Kaplan–Meier method in patients based on normal or high-total cholesterol before NAC with different intrinsic breast cancer subtype. All breast cancer (a), Luminal (b), Luminal-human epidermal growth factor receptor 2 (HER2) (c), HER2-enrich (d) and triple-negative breast cancer (e). [file 40001_2022_964_MOESM5_ESM.tif]

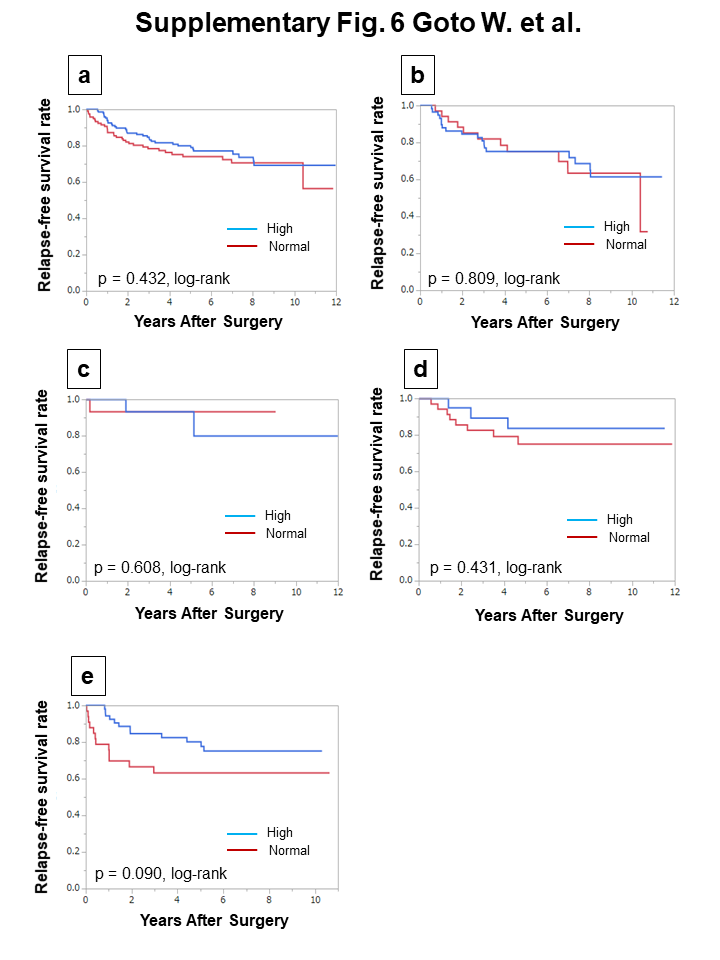

Supplement: Supplementary file 6 — Additional file 6: Fig. S6 Recurrence-free survival (RFS) using Kaplan–Meier method in patients based on normal or high-total cholesterol after NAC with different intrinsic breast cancer subtype. All breast cancer (a), Luminal (b), Luminal-human epidermal growth factor receptor 2 (HER2) (c), HER2-enrich (d) and triple-negative breast cancer (e). [file 40001_2022_964_MOESM6_ESM.tif]

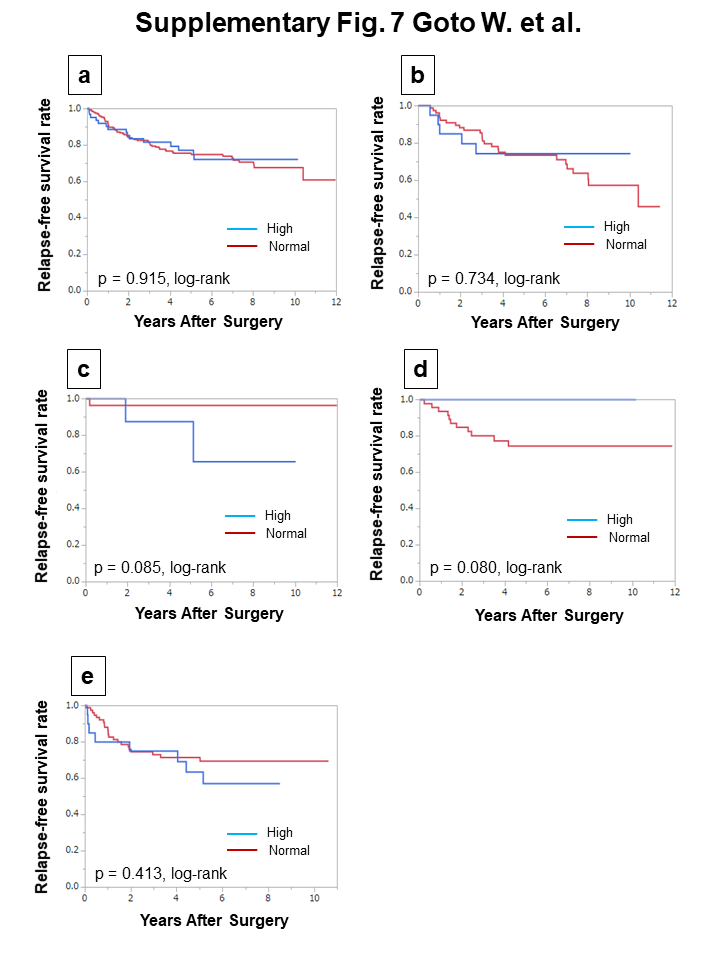

Supplement: Supplementary file 7 — Additional file 7: Fig. S7 Recurrence-free survival (RFS) using Kaplan–Meier method in patients based on normal or high-triglyceride before NAC with different intrinsic breast cancer subtype. All breast cancer (a), Luminal (b), Luminal-human epidermal growth factor receptor 2 (HER2) (c), HER2-enrich (d) and triple-negative breast cancer (e). [file 40001_2022_964_MOESM7_ESM.tif]

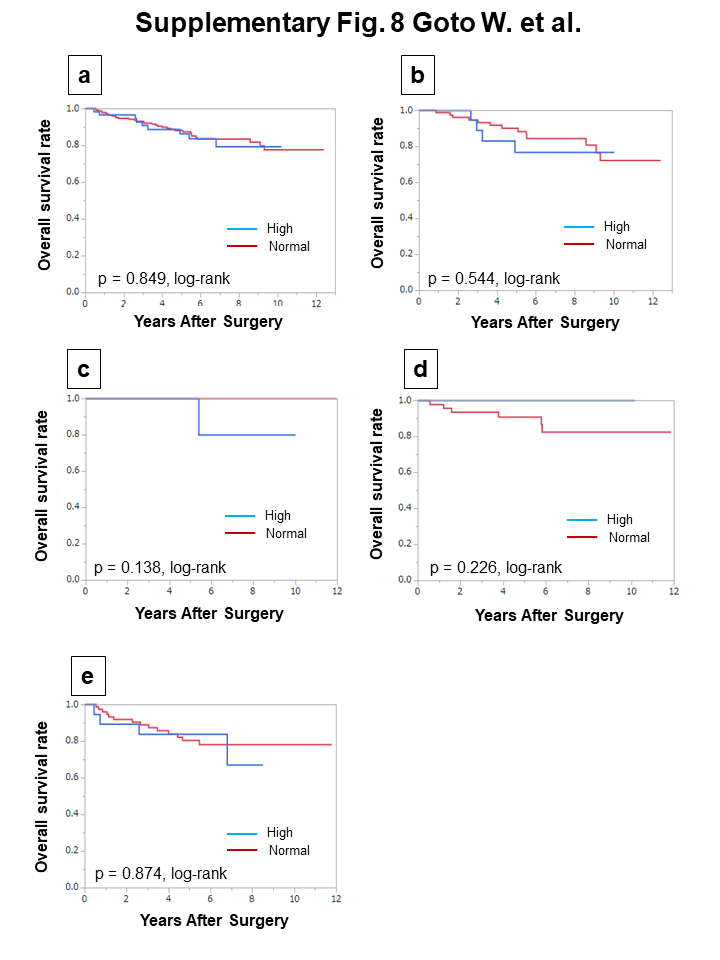

Supplement: Supplementary file 8 — Additional file 8: Fig. S8 Overall survival (OS) using Kaplan–Meier method in patients based on normal or high-triglyceride before NAC with different intrinsic breast cancer subtype. All breast cancer (a), Luminal (b), Luminal-human epidermal growth factor receptor 2 (HER2) (c), HER2-enrich (d) and triple-negative breast cancer (e). [file 40001_2022_964_MOESM8_ESM.tif]

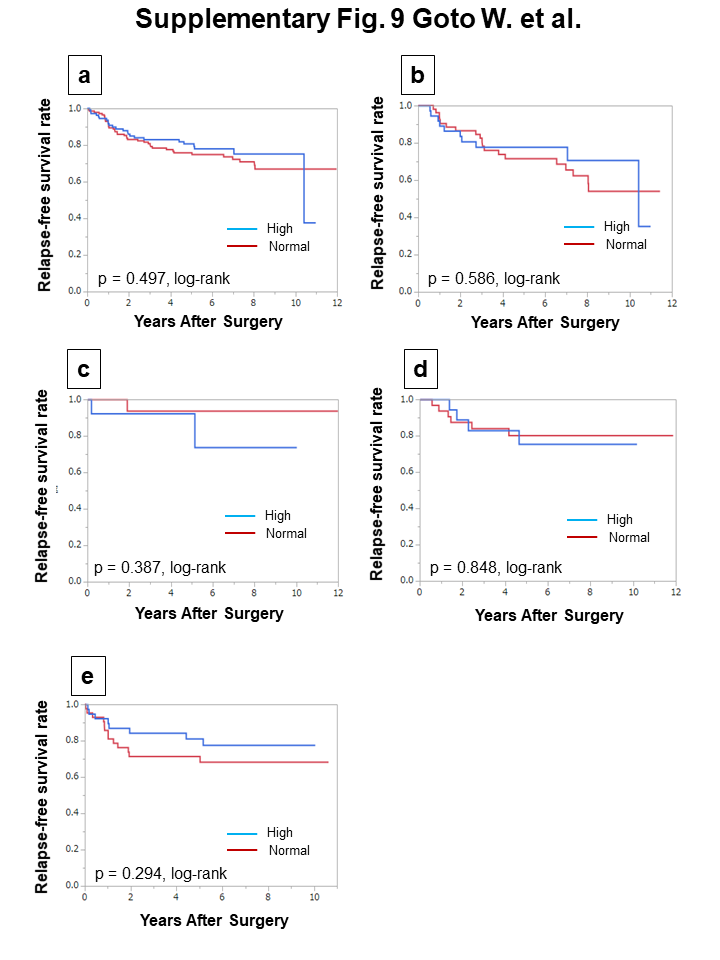

Supplement: Supplementary file 9 — Additional file 9: Fig. S9 Recurrence-free survival (RFS) using Kaplan–Meier method in patients based on normal or high-triglyceride after NAC with different intrinsic breast cancer subtype. All breast cancer (a), Luminal (b), Luminal-human epidermal growth factor receptor 2 (HER2) (c), HER2-enrich (d) and triple-negative breast cancer (e). [file 40001_2022_964_MOESM9_ESM.tif]

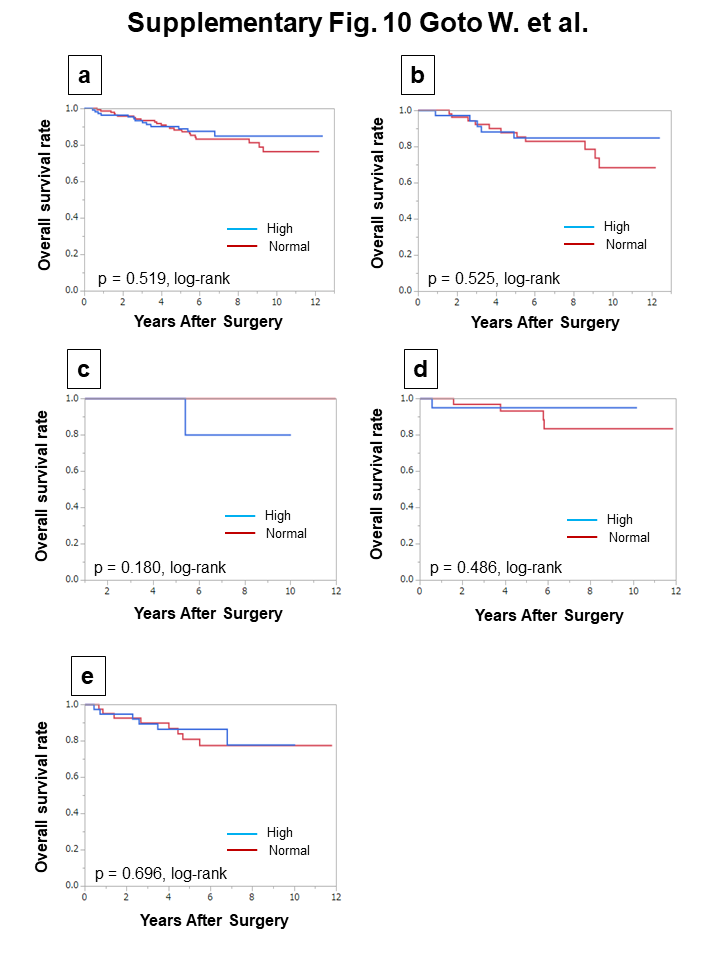

Supplement: Supplementary file 10 — Additional file 10: Fig. S10 Overall survival (RFS) using Kaplan–Meier method in patients based on normal or high-triglyceride after NAC with different intrinsic breast cancer subtype. All breast cancer (a), Luminal (b), Luminal-human epidermal growth factor receptor 2 (HER2) (c), HER2-enrich (d) and triple-negative breast cancer (e). Table S1. Relationships between immune activity and chemosensitivity. [file 40001_2022_964_MOESM10_ESM.tif]
