# Supplementary material for: Clinical verification of the relationship between serum lipid metabolism and immune activity in breast cancer patients treated with neoadjuvant chemotherapy
Source: Eur J Med Res. 2023 Jan 2;28:2. doi: 10.1186/s40001-022-00964-w (PMC9806883; doi:10.1186/s40001-022-00964-w)
Supplement: Supplementary file 11 — Additional file 11: Table S1. Relationships between immune activity and chemosensitivity. [file 40001_2022_964_MOESM11_ESM.docx]

**Supplementary Table S1. Relationships between immune activity and chemosensitivity.**

|  | pCR | | | OR | | |
| --- | --- | --- | --- | --- | --- | --- |
|  | Negative | Positive | p-value | Negative | Positive | p-value |
| All breast cancer (n = 327) | | | | | | |
| ALC (preNAC)  Low  High | 98 (47.6 %)  108 (52.4 %) | 65 (53.7 %)  56 (46.3 %) | 0.304 | 20 (62.5 %)  12 (37.5 %) | 143 (48.5 %)  152 (51.5 %) | 0.141 |
| NLR (preNAC)  Low  High | 109 (52.9 %)  97 (47.1 %) | 59 (48.8 %)  62 (51.2 %) | 0.493 | 17 (53.1 %)  15 (46.9 %) | 151 (51.2 %)  144 (48.8 %) | 0.855 |
| ALC (postNAC)  Low  High | 180 (88.2 %)  24 (11.8 %) | 113 (93.4 %)  8 (6.6 %) | 0.177 | 27 (87.1 %)  4 (12.9 %) | 266 (90.5 %)  28 (9.5 %) | 0.527 |
| NLR (postNAC)  Low  High | 76 (37.3 %)  128 (62.7 %) | 45 (37.2 %)  76 (62.8 %) | 0.991 | 16 (51.6 %)  15 (48.4 %) | 105 (35.7 %)  189 (64.3 %) | 0.117 |
| Luminal (n = 108) | | | | | | |
| ALC (preNAC)  Low  High | 36 (40.5 %)  53 (59.5 %) | 12 (63.2 %)  7 (36.8 %) | 0.081 | 7 (63.6 %)  4 (36.4 %) | 41 (42.3 %)  56 (57.7 %) | 0.211 |
| NLR (preNAC)  Low  High | 55 (61.8 %)  34 (38.2 %) | 8 (42.1 %)  11 (57.9 %) | 0.131 | 7 (63.6 %)  4 (36.4 %) | 56 (57.7 %)  41 (42.3 %) | 0.759 |
| ALC (postNAC)  Low  High | 78 (87.6 %)  11 (12.4 %) | 19 (100.0 %)  0 (0.0 %) | 0.207 | 11 (100.0 %)  0 (0.0 %) | 86 (88.7 %)  11 (11.3 %) | 0.599 |
| NLR (postNAC)  Low  High | 35 (39.3 %)  54 (60.7 %) | 5 (26.3 %)  14 (73.7 %) | 0.433 | 7 (63.6 %)  4 (36.4 %) | 33 (34.0 %)  64 (66.0 %) | 0.095 |
| Luminal-HER (n = 42) | | | | | | |
| ALC (preNAC)  Low  High | 17 (56.7 %)  13 (43.3 %) | 7 (58.3 %)  5 (41.7 %) | 0.921 | 2 (33.3 %)  4 (66.7 %) | 22 (61.1 %)  14 (38.9 %) | 0.375 |
| NLR (preNAC)  Low  High | 14 (46.7 %)  16 (53.3 %) | 9 (75.0 %)  3 (25.0 %) | 0.169 | 3 (50.0 %)  3 (50.0 %) | 20 (55.6 %)  16 (44.4 %) | 0.801 |
| ALC (postNAC)  Low  High | 25 (86.2 %)  4 (13.8 %) | 11 (91.7 %)  1 (8.3 %) | 0.616 | 3 (60.0 %)  2 (40.0 %) | 33 (91.7 %)  3 (8.3 %) | 0.104 |
| NLR (postNAC)  Low  High | 15 (51.7 %)  14 (48.3 %) | 4 (33.3 %)  8 (66.7 %) | 0.325 | 3 (60.0 %)  2 (40.0 %) | 16 (44.4 %)  20 (55.6 %) | 0.649 |
| HER2-enriched (n = 72) | | | | | | |
| ALC (preNAC)  Low  High | 12 (41.4 %)  17 (58.6 %) | 23 (53.5 %)  20 (46.5 %) | 0.346 | 1 (50.0 %)  1 (50.0 %) | 34 (48.6 %)  36 (51.4 %) | 0.968 |
| NLR (preNAC)  Low  High | 15 (51.7 %)  14 (48.3 %) | 17 (39.5 %)  26 (60.5 %) | 0.342 | 2 (100.0 %)  0 (0.0 %) | 30 (42.9 %)  40 (57.1 %) | 0.194 |
| ALC (postNAC)  Low  High | 23 (79.3 %)  6 (20.7 %) | 38 (88.4 %)  5 (11.6 %) | 0.332 | 2 (100.0 %)  0 (0.0 %) | 59 (84.3 %)  11 (15.7 %) | 0.412 |
| NLR (postNAC)  Low  High | 9 (31.0 %)  20 (69.0 %) | 17 (39.5 %)  26 (60.5 %) | 0.618 | 1 (50.0 %)  1 (50.0 %) | 25 (35.7 %)  45 (64.3 %) | 0.684 |
| TNBC (n = 105) | | | | | | |
| ALC (preNAC)  Low  High | 33 (56.9 %)  25 (43.1 %) | 23 (48.9 %)  24 (51.1 %) | 0.438 | 10 (76.9 %)  3 (23.1 %) | 46 (50.0 %)  46 (50.0 %) | 0.082 |
| NLR (preNAC)  Low  High | 25 (43.1 %)  33 (56.9 %) | 25 (53.2 %)  22 (46.8 %) | 0.331 | 5 (38.5 %)  8 (61.5 %) | 45 (48.9 %)  47 (51.1 %) | 0.562 |
| ALC (postNAC)  Low  High | 54 (94.7 %)  3 (5.3 %) | 45 (95.7 %)  2 (4.3 %) | 0.810 | 11 (84.6 %)  2 (15.4 %) | 88 (96.7 %)  3 (3.3 %) | 0.117 |
| NLR (postNAC)  Low  High | 17 (29.8 %)  40 (70.2 %) | 19 (40.4 %)  28 (59.6 %) | 0.303 | 5 (38.5 %)  8 (61.5 %) | 31 (34.1 %)  60 (65.9 %) | 0.763 |

ALC, absolute lymphocyte count. HER2, human epidermal growth factor receptor 2. NAC, neoadjuvant chemotherapy. NLR, neutrophil-to-lymphocyte ratio. OR, objective response. pCR, pathological complete response. TNBC, triple-negative breast cancer.
